# Predictors of intensive care unit admission in adult cancer patients presenting to the emergency department with COVID-19 infection: A retrospective study

**DOI:** 10.1371/journal.pone.0287649

**Published:** 2023-08-29

**Authors:** Tharwat El Zahran, Nour Kalot, Rola Cheaito, Malak Khalifeh, Natalie Estelly, Imad El Majzoub

**Affiliations:** 1 Department of Emergency Medicine, American University of Beirut Medical Center, Beirut, Lebanon; 2 Department of Internal Medicine, American University of Beirut Medical Center, Beirut, Lebanon; 3 Faculty of Medicine, American University of Beirut, Beirut, Lebanon; 4 Sheikh Shakhbout Medical City, Abu Dhabi, United Arab Emirates; Stanford University School of Medicine, UNITED STATES

## Abstract

**Background:**

Adult cancer patients with COVID-19 were shown to be at higher risk of Intensive Care Unit (ICU) admission. Previously published prediction models showed controversy and enforced the importance of heterogeneity among different populations studied. Therefore, this study aimed to identify predictors of ICU admission (demographic, clinical, and COVID-19 targeted medications) in cancer patients with active COVID-19 infection presenting to the Emergency Department (ED).

**Methods:**

This is a retrospective cohort study. It was conducted on adult cancer patients older than 18 years who presented to the American University of Beirut Medical Center ED from February 21, 2020, till February 21, 2021, and were found to have COVID-19 infection. Relevant data were extracted from electronic medical records. The association between different variables and ICU admission was tested. Logistic regression was done to adjust for confounding variables. A p-value less than 0.05 was considered significant.

**Results:**

Eighty-nine distinct patients were included. About 37% were admitted to the ICU (n = 33). Higher ICU admission was seen in patients who had received chemotherapy within one month, had a respiratory rate at triage above 22 breaths per minute, oxygen saturation less than 95%, and a higher c-reactive protein upon presentation to the ED. After adjusting for confounding variables, only recent chemotherapy and higher respiratory rate at triage were significantly associated with ICU admission.

**Conclusion:**

Physicians need to be vigilant when taking care of COVID-19 infected cancer patients. Patients who are tachypneic at presentation and those who have had chemotherapy within one month are at high risk for ICU admission.

## Background

One of the most vulnerable groups of patients to critical illness from respiratory viral infections are cancer patients [1]. It is postulated that patients with cancer who are infected with the SARS-CoV-2 Coronavirus may have worse outcomes than others [2]. Published work reported higher morbidity and mortality rates from COVID-19 among cancer patients compared to their cancer-free counterparts [2–6].

Admission to the Intensive Care Units (ICU) plays a significant role in the management of COVID-19 patients, with some reports showing a reduced mortality rate among those admitted to critical care units [7–9]. As a result, several studies developed prediction models and risk scores for ICU admission in COVID-19. Nevertheless, these studies have shown various results that were sometimes controversial in terms of the effect of the type of cancer and its therapies on the prognosis of infected patients [10]. This controversy might be due to a composite of causes, including methodological differences, regional care differences, SARS-CoV-2 variants, heterogeneity of the evaluated population, as well as the large heterogeneity embedded within the cancer and COVID-19 diseases [10].

During the COVID-19 pandemic, emergency departments (EDs) have been on the frontlines, playing an essential role in detecting infected patients, providing urgent medical care [11], and deciding on the proper disposition of patients. These departments have been challenged and overwhelmed by the increasing number of COVID-19 cases worldwide. Consequently, it has been of utmost importance in ED settings to be able to predict which cancer patients with COVID-19 are at risk of deteriorating and having worse outcomes. The knowledge of these predictors can be used to assure a proper and timely risk stratification, adjust management accordingly, avoid ICU admission delay [12], and prioritize the admission of high-risk patients.

To date, no studies have been conducted in Lebanon to determine the predictors of ICU admission in cancer patient population with COVID-19 in Lebanon. The objective of the present study is to identify predictors of ICU admission (e.g., demographic, clinical, and COVID-19 targeted medications) in cancer patients with active COVID-19 infection presenting to the ED. This will contribute to risk stratification of this population, optimize the medical management, and potentially help in developing a predictive tool to identify COVID19-positive cancer patients who are at a higher risk of complications leading to mortality.

## Methodology

### Study design and setting

This is a retrospective cohort study conducted at the American University of Beirut Medical Center (AUBMC), a tertiary care academic hospital in Lebanon. The study enrolled all cancer patients who presented from February 21, 2020, till February 21, 2021, to the ED of AUBMC and were diagnosed with COVID-19 infection. The ED is run as a closed unit by onsite coverage of emergency medicine specialists 24 hours a day, seven days a week. The study was approved by the Institutional Review Board (IRB) at AUBMC under the protocol number (BIO-2021-0015). Informed consent was waived given the retrospective nature of the study. In order to protect patients’ information and confidentiality, subjects’ names were not collected. Each patient was anonymously assigned a study ID in the data collection sheet. The patient’s study ID were kept on a separate log sheet and were only accessible by the primary investigator and the research coordinator.

### Study population

Patients included were adult (>18 years old) cancer patients who presented to the ED of AUBMC from February 21, 2020, till February 21, 2021, and were positive for COVID-19 infection. We defined COVID-19 infection as a positive result of the SARS-COV-2 nucleic acid RT-PCR test using the nasal swab samples.

Patients not fitting any of the above criteria, as well as those who presented dead on arrival to the ED, were excluded.

### Data collection and sampling

Eligible patients were identified through the electronic health system (Epic Systems, Verona, WI, USA). Following an IRB-approved unified data collection manual adjusted after a pilot data collection, the two team members who retrieved the data used the same nomenclature, definitions, and workflow. Study data were collected and managed using REDCap (Research Electronic Data Capture) a secure web-based application designed to support data capture for research studies that is Health Insurance Portability and Accountability Act compliant [13, 14] A quality control was reviewed by one team member down the line.

The data collection form was divided into multiple sections (S1 Dataset). The first section encompassed the demographic and medical history (smoking status, medication, and comorbidities) of the patients. It also included a subsection about the cancer history of the patient (type of cancer, its spread, and treatment modalities, including chemotherapy and immunotherapy). The second section was about the details of the ED visit where the patient was confirmed to be COVID-19 infected. In addition, we collected information about vital signs, treatment given in the ED, and ED disposition. Finally, the third section was about the patients’ hospital stay, which included all complications (sepsis, acute kidney injury (AKI), cardiac and respiratory complications (e.g., acute respiratory distress syndrome, ARDS and pulmonary embolism, PE)) along with the procedures done (central line or chest tube insertion, dialysis, tracheostomy) and hospital discharge date, and disposition.

### Statistical analysis

Statistical analysis was performed using SPSS version 25.0 (Armonk, NY: IBM Corp). Categorical variables were described using frequencies and percentages. Continuous variables were reported using means, and standard deviations.

The dependent variable was ICU admission versus no ICU admission. The association between different variables and ICU admission was tested using Pearson’s Chi-square or Fisher’s exact test, Student’s *t-*test and Mann Whitney U where appropriate.

Later, logistic regression was done to adjust for confounding variables and identify factors that were associated with ICU admissions in these patients. We included variables with p-value less than 0.2. Variable(s) entered in step 1: Vasopressors, Remdesivir, Tocilizumab, Steroid, Antibiotics, Anticoagulant, CRP, RR at triage (reference ≤ 22), O2 at triage (reference ≥ 95 mmHg), Chemotherapywithin1monthofpresentation. A p-value less than 0.05 was considered significant.

## Results

### 1. Demographics and clinical characteristics of COVID-19 cancer patients

A total of 89 cancer COVID-19 infected patients were included in the study. Their average age was 66 years (± 13.6). The majority were males (64%) and with solid cancer (74.2%). About half of them were smokers (52.8%) and underwent chemotherapy within 1 month of presentation (52.8%). Only 6 patients did bone marrow transplants (BMT) within 1 year of presentation. Hypertension was the main comorbidity among patients (39.3%), followed by cardiovascular diseases (25.8%), dyslipidemia (23.6%), and diabetes mellitus (14.6%). Only 4 of the patients were on baseline steroids before the ED visit. More than a third of the patients in our sample were admitted to the ICU (n = 33). Their mean age was 67 years (±11.2) and were mainly males (69.7%). (Table 1)

**Table 1:** Association of baseline characteristics of oncology COVID-19 patients with ICU admission.

Characteristics		Total N = 89	No ICU n = 56 (63%)	ICU n = 33 (37%)	p-value	OR	95% CI
**Age (years)**		66.3 (13.6)	65.9 (14.8)	67 (11.2)	0.711		
**Sex**	**Female**	32 (36%)	22 (39.3%)	10 (30.3%)	0.394	Ref	
	**Male**	57 (64%)	34 (60.7%)	23 (69.7%)		1.488	0.596–3.719
**History of smoking**		47 (52.8%)	28 (50%)	19 (57.6%)	0.489	1.357	0.571–3.228
**Type of Cancer**	**Liquid**	23 (26.7%)	14 (25.5%)	9 (29%)	0.801	Ref	
	**Solid**	63 (73.3%)	41 (74.5%)	22 (71%)		0.835	0.312–2.234
**Metastatic tumor**		34 (52.3%)	20 (47.6%)	14 (60.9%)	0.306	1.711	0.609–4.809
**Bone Marrow Transplant within 1 year**		6 (6.8%)	3 (5.4%)	3 (9.4%)	0.664	1.828	0.346–9.642
**Chemotherapy within 1 month**		47 (52.8%)	26 (46.4%)	21 (63.6%)	0.116	2.019	0.835–4.88
**Immunotherapy**		19 (21.3%)	12 (21.4%)	7 (21.2%)	0.981	0.987	0.345–2.823
**Comorbidities**	**Cardiovascular Diseases**	23 (25.8%)	17 (30.4%)	6 (18.2%)	0.205	0.51	0.178–1.46
	**Diabetes Mellitus**	13 (14.6%)	6 (10.7%)	7 (21.2%)	0.219	2.244	0.683–7.367
	**Hypertension**	35 (39.3%)	22 (39.3%)	13 (39.4%)	0.992	1.005	0.416–2.423
	**Dyslipidemia**	21 (23.6%)	14 (25%)	7 (21.2%)	0.684	0.808	0.288–2.264
	**Cerebrovascular accident/TIA**	2 (2.2%)	1 (1.8%)	1 (3%)	1	1.719	0.104–28.43
	**Chronic Obstructive Pulmonary Disease**	8 (9%)	7 (12.5%)	1 (3%)	0.249	0.219	0.026–1.863
	**Chronic Kidney Disease**	16 (18%)	10 (17.9%)	6 (18.2%)	0.969	1.022	0.334–3.127
	**Hemiplegia**	1 (1.1%)	1 (1.8%)	0 (0%)	1		
	**Peptic ulcer disease**	2 (2.2%)	1 (1.8%)	1 (3%)	1	1.719	0.104–28.43
	**Liver Disease**	3 (3.4%)	1 (1.8%)	2 (6.1%)	0.552	3.548	0.309–40.73
	**Other** *	58 (65.2%)	40 (71.4%)	18 (54.5%)	0.106	0.48	0.196–1.178

Data are presented as numbers with percentages.

The P-value for the difference between two adjacent columns is calculated by chi-square or Fisher’s exact test where appropriate.

Abbreviations: OR: odds ratio, 95%CI: 95% Confidence Interval, Ref = Reference, ICU = intensive care unit, ED = emergency department

*Other comorbidities are thyroid disease, psychiatric disorders, and rheumatologic diseases.

Most of the patients had tachycardia (n = 79, 89.8%) and 40.4% had low oxygen saturation at triage inferior to 95mmHg (n = 36, 40.4%). (Table 2)

**Table 2:** Association of vital signs and ED treatment of COVID-19 oncology patients with ICU admission.

		Total n = 89	No ICU n = 56 (63%)	ICU n = 33 (37%)	p value	OR	95%CI
**ED treatment**							
**Mechanical Ventilation in ED**		11 (12.4%)	0	11 (33.3%)	< .001		
**Vasopressors**		7 (7.9%)	1 (1.8%)	6 (18.2%)	0.01	12.222	1.4–106.674
**Steroids**		50 (56.2%)	26 (46.4%)	24 (72.7%)	0.016	3.077	1.215–7.789
**Antibiotics**		43 (48.3%)	31 (55.4%)	12 (36.4%)	0.083	0.461	0.19–1.115
**Anticoagulants**		42 (47.2%)	22 (39.3%)	20 (60.6%)	0.052	2.378	0.986–5.735
**Plasma**		6 (6.7%)	4 (7.1%)	2 (6.1%)	1	0.839	0.145–4.849
**Remdesivir**		17 (19.1%)	13 (2 3.2%)	4 (12.1%)	0.198	0.456	0.135–1.539
**Ivermectin**		13 (14.6%)	7 (12.5%)	6 (18.2%)	0.54	1.556	0.475–5.099
**Tocilizumab**		8 (9%)	2 (3.6%)	6 (18.2%)	0.048	6	1.134–31.735
**Baricitinib**		3 (3.4%)	1 (1.8%)	2 (6.1%)	0.552	3.548	0.309–40.73
**Vital Signs**							
**Heart rate at triage**	**< = 100**	46 (51.7%)	32 (57.1%)	14(42.4%)	0.180	Ref	
	**>100**	43(48.3%)	24(42.9%)	19(57.6%)		1.180	0.758–4.319
**Systolic blood pressure at triage**	**< = 100**	9 (10.2%)	7 (12.5%)	2 (6.3%)	0.478	Ref	
	**>100**	79 (89.8%)	49 (87.5%)	30 (93.8%)		2.143	0.417–11.001
**Respiratory rate at triage**	**< = 22**	72 (83.7%)	53 (94.6%)	19 (63.3%)	0.001	0.098	0.025–0.389
	**>22**	14 (16.3%)	3 (5.4%)	11 (36.7%)		Ref	
**Temperature (**°**C) at triage**	**<37.5**	50 (57.5%)	30 (53.6%)	20 (64.5%)	0.323	Ref	
	**> = 37.5**	37 (42.5%)	26 (46.4%)	11 (35.5%)		0.635	0.257–1.567
**Oxygen Saturation level (mmHg)**	**SpO2 <95**	36 (40.4%)	16 (28.6%)	20 (60.6%)	0.003	3.846	1.552–9.523
	**SpO2 > = 95**	53 (59.6%)	40 (71.4%)	13 (39.4%)		Ref	0.105–0.644

Data are presented as numbers with percentages.

The p-value for the difference between two adjacent columns is calculated by chi-square or Fisher’s exact test where appropriate.

Abbreviations: OR: odds ratio, 95%CI: 95% Confidence Interval, Ref = Reference, SpO2 = Oxygen saturation, ICU = intensive care unit, ED = emergency department

Patients with liquid or solid tumors were homogenous in terms of age, smoking status, and presence of comorbidities. However, patients with liquid tumors were mainly males (95.7%, p<0.001) and had more moderate to severe kidney diseases (34.8%, p = 0.021).

### 2. Treatments and health-related complications of COVID-19 cancer patients

In the ED, patients were treated mainly with steroids (56.2%), antibiotics (48.3%), and anticoagulants (47.2%). They were also treated with Remdesivir (19.1%), Ivermectin (14.6%), Tocilizumab (9%), or convalescent plasma (6.7%). Only 7.9% of patients were treated with vasopressors (n = 7). (Table 2)

During their hospital stay, patients faced complications whom 33.7% developed respiratory complications, including ARDS, pneumothorax, or respiratory failure, while 15.7% had septic shock, and 7.9% developed cardiovascular complications. Only 9% of patients required dialysis (n = 8). About 28.1% required endotracheal intubation either in the ED or during their hospital stay (n = 25). The average length of hospital stay was 30.7 days.

#### 2.1 Characteristics of patients who required intubation in the ED

Eleven patients were intubated in the ED (12.4%). There was no significant difference in gender, age, smoking status, and presence of comorbidities between patients who were endotracheal intubated in the ED and those who were not. The average age of intubated patients was 66.7 years (±10.2) and were mainly males (81.8%).

For vital signs, patients who were intubated in the ED more frequently had an oxygen saturation < 95%, tachypnea with a RR >22 breaths/minute (72.7% vs. 8%, p < .001), or tachycardia (Heart Rate>100 beats/minute) (81.8% vs. 43.6%, p = 0.018).

Patients who were intubated were more on Ivermectin (36.4% vs. 11.5%, p = 0.051), vasopressors (54.5% vs. 1.3%, p<0.001), or anticoagulants (81.8% vs. 42.3%, p = 0.014). Intubated patients were less on antibiotics (9.1% vs. 53.8%, p = 0.005). The c-reactive protein (CRP) level was significantly higher in intubated patients (187.5 ±93.3 vs. 85.5±74.6, p<0.001).

#### 2.2 Characteristics of patients who had respiratory complications

About 30 patients developed respiratory complications (33.7%), including pneumothorax, acute respiratory distress syndrome, and respiratory failure. However, the presence of respiratory complications was not significantly impacted by gender, age, smoking status, or presence of comorbidities.

For vital signs, patients with respiratory complications had significantly lower oxygen saturation level at triage less than 95 mmHg (56.7% vs. 32.2%, p = 0.026) or tachypnea RR superior to 22 (35.7% vs. 6.9%, p = 0.001).

Patients with respiratory complications were using significantly more Tocilizumab (20% vs. 3.4%, p = .016), steroids (76.7% vs. 45.8%, p = 0.005) or anticoagulants (66.7% vs. 37.3%, p = 0.009). They had significantly elevated CRP level (132.8 vs. 82.6 p = 0.011). They were also more admitted to the ICU (75.8% vs. 13.6%, p<0.001), with a higher mortality (23.3% vs. 1.7%, p = 0.002).

### 3. Predictors of ICU admission in COVID-19 cancer patients

None of the baseline characteristics, including gender, age, smoking status, and presence of comorbidities significantly associated with ICU admission (p > 0.05). (Table 1)

Patients in ICU were significantly using more vasopressors (18.2% vs. 1.8%, p = 0.01) and were more mechanically ventilated in the ED (p<0.001) than patients who were not admitted to the ICU. They were also significantly 6 times more on Tocilizumab (18.2% vs. 12.5%, p = 0.048) and 3 times more on steroids (72.7% vs. 46.4%, p = 0.016). (Table 2)

For vital signs, low oxygen saturation level at triage <95 mmHg (60.6% vs. 28.6%, p = .003) and elevated respiratory rate (> 22 breaths/min) (36.7% vs. 5.4%, p = 0.001) were significantly associated with ICU admission. However, there was no significant difference in systolic blood pressure and temperature of patients who were admitted to the ICU compared to patients who were not admitted to the ICU (p > 0.05). (Table 2)

The CRP level upon ED presentation was significantly higher in patients admitted to ICU than in patients who did not require an ICU admission (140.8 ±98.2 vs. 76.1 ±65.9, p = 0.003). (Table 3)

**Table 3:** Association of ED laboratory data of COVID-19 oncology with ICU admission.

Laboratory Data	Total N = 89	No ICU n = 56 (63%)	ICU n = 33 (37%)	p-value
**White blood cells. count**	8735.830 (11719.0215)	7548.5 (7071.2)	10714.7 (16808.96)	0.31
**Absolute Neutrophil Count**	5720.476 (4285.4441)	5558.5 (4160.7)	5997.4 (4547.26)	0.653
**Hemoglobin**	11 (1.9456)	11.1 (1.9)	10.8 (2.1)	0.393
**Platelets**	184323.864 (92905.1327)	178514.55 (86596.1)	194006.1 (103234.996)	0.452
**Lactate Dehydrogenase**	568.77 (560.581)	658.8 (738.7)	465.3 (204.2)	0.239
**Lactic acid Venous**	1.9024 (1.39752)	1.8 (1.7)	2.1 (0.82)	0.445
**C-Reactive Protein**	99.5 (84.5)	76.1 (65.9)	140.8 (98.2)	0.003
**d-dimer**	1379.9 (3418.1)	944 (1061.9)	2142.7 (5482.1)	0.3
**Procalcitonin**	1 (2.9)	0.8 (3.5)	1.2 (1.8)	0.552
**Troponin T**	0 (0.1)	0 (0.1)	0 (0)	0.539

Data are presented as mean with standard deviation.

The p-value for the difference between two adjacent columns is calculated by the T-test.

Additionally, patients admitted to ICU significantly develop more respiratory complications (75.8% vs. 8.9%, p <0.001), AKI (42.4% vs. 7.1%, p<0.001), PE (p = 0.048), septic shock (p<0.001). They were significantly more on dialysis (21.2% vs. 1.8%, p = 0.004) and more died (p<0.001).

#### 3.1 Predictors Of ICU admission in COVID-19 cancer patients using logistic regression (Table 4)

After adjusting for confounding variables using logistic regression, Remdesivir (aOR = 0.05, 95%, CI = 0.005–0.463) and antibiotics (aOR = 0.15, 95%, CI = 0.031–0.73) were found to reduce the risk of ICU admission. RR > 22 in triage was significantly associated with ICU admission (aOR = 17.431, 95%CI = 2.4–125.1). Patients admitted to ICU were more on steroids (aOR = 13.4, 95%CI = 2.3–78.2) and more on Tocilizumab (aOR = 18.5, 95%CI = 1.9–179.6). They had also significantly received more chemotherapy within 1 month of presentation (aOR = 5.5, 95%CI = 1.2–25.8). (Table 3)

**Table 4:** Logistic regression: Factors associated with mortality in COVID-19 ICU patients.

	p-value	aOR	95% C.I.	
Remdesivir	0.008	0.05	0.005	0.463
Tocilizumab	0.012	18.481	1.902	179.595
Steroids	0.004	13.399	2.297	78.159
Antibiotics	0.019	0.15	0.031	0.73
RR at triage	0.004	17.431	2.429	125.111
Chemotherapy within 1 month of presentation	0.029	5.545	1.193	25.78

Variable(s) entered in step 1: Vasopressors, Remdesivir, Tocilizumab, Steroid, Antibiotics, Anticoagulant, CRP, RR at triage (reference ≤ 22), O2 at triage (reference ≥ 95 mmHg), Chemotherapywithin1monthofpresentation.

Omnibus < .001, R2 = .577, Hosmer = 0.918

95%C.I.: 95% Confidence Interval, aOR: adjusted Odds Ratio

## Discussion

The present study aims to detect the predictors of ICU admission for adult COVID-19 patients with cancer who present to the ED. ICU admission risk for cancer patients who presented to the ED with COVID-19 infection was significantly associated with chemotherapy within one month, a respiratory rate at triage above 22 breaths per minute, oxygen saturation less than 95%, and a higher CRP. Out of these, after multivariate analysis, only high respiratory rate and recent chemotherapy were top predictors of ICU admission. Of note, ICU admission risk in ED for cancer patients infected with COVID-19 was not significantly associated with the included sample’s demographic variables.

Our study focuses on cancer outpatients who present to the ED for COVID-19 infection, aiming to aid the ED staff in establishing a better specific assessment and management of patients. Furthermore, this is the first study done in Lebanon to evaluate the morbidity of COVID-19 in cancer patients.

Studies, including ours, which evaluated the role of recent chemotherapy on COVID-19 outcomes, have shown controversial results. Zhang et al. showed that rates of severe respiratory COVID-19 were associated with recent chemotherapy [15]. On the contrary, Jee et al. found that cytotoxic chemotherapy administered between 90 and 14 days before testing positive for COVID-19 has no increased rate for ICU admission [16], which is consistent with previous data. Such controversy in results may be explained by the high heterogeneity of chemotherapy drugs that differ in their mechanisms. Interestingly, some agents were found to have anti-cytokine storm effects, which have shown promise in patients with COVID-19 (e.g., the Janus kinase (JAK) inhibitors and Bruton’s tyrosine kinase (BTK) inhibitors) [17, 18]. These antineoplastic drugs revealed the ability to prevent the cytokine storm generation thus suppressing the immune system response along with multiple organ failure [19]. Noteworthy, none of our patients were receiving these drugs. Another explanation for the contradictory results regarding chemotherapy could be due to different study models that have not accounted for factors that may affect the results [16].

Patients presenting to the ED with a respiratory rate exceeding 22 breaths per minute (tachypnea) was a top predictor for ICU admission. Respiratory rate changes are an important marker often preceding major complications, including respiratory depression, and failure [20]. Since COVID-19 has the potential to affect the respiratory system [21], changes in resting respiratory rate might occur in the early stages of infection [22]. High respiratory rates displayed the ability to predict most in-hospital cardiac arrests as well as admission to the ICU [23]. When compared to heart rate, respiratory rate is found to be a better indicator of the patient’s stability [24]. Furthermore, Subbe et. al showed that respiratory rate is superior not only to pulse rate but also to both blood pressure in detecting high-risk patient groups [25].

In addition, vital signs are essential to monitor the patient overall status. Oxygen saturation, compared to the invasive arterial blood-gas measurement, serves as a more accessible indicator of the oxygenation for triage purposes [26]. The univariate analysis of this study showed that an oxygen saturation less than 95% at presentation to ED was significantly associated with ICU admission. Akhavan et al. found that lower ambulatory oxygen saturation was strongly correlated with requiring high oxygen supplementation and mechanical ventilation among admitted ED COVID-19 patients [27]. Severe respiratory failure and death associated with coronavirus infection may be the result of damaged alveoli and edema formation, which hinders the lung’s ability to oxygenate the blood, as reflected in reduced oxygen saturation [28, 29].

The CRP level was significantly higher in COVID-19 cancer patients admitted to ICU which is consistent with Wang et al. findings. Higher CRP levels were associated with aggravated COVID-19 cases, and these levels occurred before disease progression [30]. C-reactive protein is a well-known marker of systemic inflammation and severe infection [31]. In COVID-19 infection, CRP was established as an independent outcome predictor as well as an independent discriminator of the severity of the disease [32–35]. High levels of CRP were considered the most important predictor of COVID-19 severity in cancer patients [36]. Of note, especially when looking at the multivariate analysis of our study, high CRP does commonly occur in cancer patients, which implies that it might be questionable whether or not it should be considered to be an independent prognostic factor in cancer COVID-19 patients [37].

As in other studies, we found that Remdesivir displayed potential benefits in terms of reducing the risk of ICU admission. When prescribed alone to cancer patients with COVID-19, this drug was associated with a reduced 30-day all-cause mortality (aOR, 0.41; 95% CI: 0.17–0.99) [38]. This nucleotide analog ribonucleic acid (RNA) polymerase inhibitor has shown promising results. In a cohort of severe COVID-19 patients, clinical improvement was observed in 68% of 53 patients [39]. In a double-blind, randomized, placebo-controlled trial in hospitalized adults with COVID-19, intravenous Remdesivir was shown to significantly speed the time to improvement versus placebo (p < 0.001) [40]. Moreover, Remdesivir usage in treating outpatients with mild to moderate COVID-19 was approved by the US Food and Drug Administration which also supports its efficacy [41].

While the literature suggests that early antibiotic administration in COVID-19 cases has no impact on mortality rates [42] and can increase the risk of adverse outcomes [43], we found that it is significantly associated with a lower risk of ICU admission of infected cancer patients. Large multi-centric studies are urgently required to better assess this association [44].

Large multi-centered studies are also needed to investigate the impact of other treatments, including Tocilizumab and steroids on the morbidity and mortality of cancer patients with COVID-19. While limited evidence is available on treatment with Tocilizumab for COVID-19 [45], data on steroids’ impact is conflicting [38, 46]. In our study population, these drugs were given by ED physicians to patients who were considered to have a more severe status. This may account for treatment options being associated with increased ICU admission.

## Limitations

The present study had several limitations. First, the included sample size was small, and the study was retrospectively done in a single tertiary care center. However, AUBMC has the largest cancer center in Lebanon for cancer patients and treats patients from all over the Middle East and North Africa (MENA) region. Another limitation is the evolving nature of the COVID-19 virus and its variants and the discovery of new effective treatment methods along with vaccination that would affect our observations.

## Conclusion

In conclusion, we found that patients who have received chemotherapy within one month of the infection or whose RR at triage exceeds 22 breaths per minute are significantly at greater risk of requiring ICU admission. Higher CRP level, requiring increased use of steroids and Tocilizumab were associated with aggravated COVID-19 cases. Remdesivir displayed potential benefits in terms of reducing the risk of ICU admission. Finally, early antibiotic administration was significantly associated with a lower risk of ICU admission of infected cancer patients. ED physicians should be vigilant when treating cancer patients with COVID-19 and look for predictors of worsening and start prompt therapy early on.

## Supporting information

S1 Checklist STROBE-checklist cancer icu study. (DOC)

S1 Dataset Covid oncology master dataset. (XLSX)
